# More Than Meets the Eye: Associations of Vaginal Bacteria with Gram Stain Morphotypes Using Molecular Phylogenetic Analysis

**DOI:** 10.1371/journal.pone.0078633

**Published:** 2013-10-24

**Authors:** Sujatha Srinivasan, Martin T. Morgan, Congzhou Liu, Frederick A. Matsen, Noah G. Hoffman, Tina L. Fiedler, Kathy J. Agnew, Jeanne M. Marrazzo, David N. Fredricks

**Affiliations:** 1 Vaccine and Infectious Disease Division, Fred Hutchinson Cancer Research Center, Seattle, Washington, United States of America; 2 Public Health Science Division, Fred Hutchinson Cancer Research Center, Seattle, Washington, United States of America; 3 Department of Laboratory Medicine, University of Washington, Seattle, Washington, United States of America; 4 Department of Obstetrics and Gynecology, University of Washington, Seattle, Washington, United States of America; 5 Department of Medicine, University of Washington, Seattle, Washington, United States of America; 6 Department of Microbiology, University of Washington, Seattle, Washington, United States of America; Columbia University, United States of America

## Abstract

Bacterial vaginosis (BV) is a highly prevalent condition associated with adverse health outcomes. Gram stain analysis of vaginal fluid is the standard for confirming the diagnosis of BV, wherein abundances of key bacterial morphotypes are assessed. These Lactobacillus, *Gardnerella*, *Bacteroides*, and *Mobiluncus* morphotypes were originally linked to particular bacterial species through cultivation studies, but no studies have systematically investigated associations between uncultivated bacteria detected by molecular methods and Gram stain findings. In this study, 16S-rRNA PCR/pyrosequencing was used to examine associations between vaginal bacteria and bacterial morphotypes in 220 women with and without BV. Species-specific quantitative PCR (qPCR) and fluorescence in Situ hybridization (FISH) methods were used to document concentrations of two bacteria with curved rod morphologies: *Mobiluncus* and the fastidious BV-associated bacterium-1 (BVAB1). Rank abundance of vaginal bacteria in samples with evidence of curved gram-negative rods showed that BVAB1 was dominant (26.1%), while *Mobiluncus* was rare (0.2% of sequence reads). BVAB1 sequence reads were associated with *Mobiluncus* morphotypes (p<0.001). Among women with curved rods, mean concentration of BVAB1 DNA was 2 log units greater than *Mobiluncus* (p<0.001) using species-specific quantitative PCR. FISH analyses revealed that mean number of BVAB1 cells was 2 log units greater than *Mobiluncus* cells in women with highest Nugent score (p<0.001). *Prevotella* and *Porphyromonas* spp. were significantly associated with the “Bacteroides morphotype,” whereas *Bacteroides* species were rare. Gram-negative rods designated *Mobiluncus* morphotypes on Gram stain are more likely BVAB1. These findings provide a clearer picture of the bacteria associated with morphotypes on vaginal Gram stain.

## Introduction

Bacterial vaginosis (BV) is a dysbiotic condition found in up to 29% of women in the United States when using Gram stain to define BV [1]. The type of vaginal bacterial community a woman harbors can have implications for her health and her newborn infant. BV has been consistently associated with serious reproductive and health sequelae such as increased risk for preterm birth [2], and sexually transmitted diseases including HIV acquisition [3] and transmission [4]. Although BV responds to antibiotics, recurrence rates are high [5]. Clinically, BV is often diagnosed using Amsel’s criteria, a set of four signs or laboratory observations [6]. The gold standard for diagnosis of BV in research settings is Gram staining of vaginal fluid with microscopic evaluation of bacterial morphologies and abundances using well validated scoring systems [7,8]. No studies have systematically examined associations between vaginal bacteria described using high-resolution molecular methods and bacterial morphologies seen on Gram stain.

The Gram stain approach for BV diagnosis defined by Nugent et al. evaluates bacterial morphologies and abundances using a standardized weighted scoring system resulting in scores from 0-10 [8]. Scores of 0-3 denote healthy microbiota, with the presence of Gram-positive rods designated Lactobacillus morphotypes. Scores of 4-6 denote intermediate microbiota, and 7-10 BV-like microbiota. Scores of 7-8 indicate increased abundance of Gram-negative or Gram-variable rods or coccobacilli, designated *Gardnerella* and/or *Bacteroides* morphotypes. Women with Nugent scores 9-10 have detection of curved Gram-negative rods designated *Mobiluncus* morphotypes. In this scheme, bacterial morphotypes serve as surrogates for the representation of putative bacterial species present in vaginal fluid. Typically, bacteria need to be present at 10^5^ CFU/mL for detection by Gram stain [9]. Although diagnosis of BV by Nugent score is not a point-of-care approach due to the need for a highly trained microscopist with experience reading vaginal smears, it has the advantages of simple sample collection, efficient storage and transport, a standardized scale for interpretation, and reliability [8]. Recent molecular methods have revealed novel, uncultivated bacteria in women with BV [10-13]. We ask whether bacteria other than the ones previously described (Lactobacillus, *Gardnerella*, *Bacteroides* and *Mobiluncus*) contribute to bacterial morphotypes observed by Gram stain. 

Fluorescence in situ hybridization (FISH) of vaginal fluid has demonstrated at least two bacteria have curved rod morphologies; one designated “BV-associated bacterium-1” (BVAB1), and the other *Mobiluncus* [10]. This observation prompted us to ask whether some curved rod morphotypes seen in Gram stains of vaginal fluid are BVAB1 rather than *Mobiluncus* species. Given the widespread use of the Gram stain technique to describe the vaginal microbiota and to diagnose BV [14-16], it is imperative to understand bacteria associated with each morphotype, so that misattribution does not lead to false conclusions about the role of particular bacterial species in BV. For example, Gram stain results have been used in several studies to assess BV treatment outcomes and relapse [14,15,17]. Nyirjesy et al. found that a clindamycin-based regimen was more effective in reducing the abundance of *Mobiluncus* morphotypes, which also correlated with a higher BV cure rate [14]. In another study of bacterial interactions in the vagina, *in vitro* susceptibility tests were conducted on *Bacteroides fragilis* and this bacterium was found not susceptible to the antibacterial activity of the lactobacilli tested [18]. Cultivation-independent methods have detected few *Bacteroides* or *Mobiluncus* in women with BV, both key Gram stain morphotypes [10,19]. In this study, we systematically investigated associations between vaginal bacteria described by PCR/high-resolution phylogenetic analysis, and bacterial morphotypes observed by Gram stain. 

## Methods

### Ethics statement

Vaginal samples were collected using Protocol #1789 which was approved by the Institutional Review Board (IRB) at Fred Hutchinson Cancer Research Center (IR# 5485). All study participants provided written informed consent prior to enrollment in the study. Consent forms were also approved by the IRB as part of Protocol #1789.

### Study population and sample collection

The study population comprised 220 women seen at the Public Health, Seattle and King County Sexually Transmitted Diseases Clinic (STD Clinic) between September 2006 and June 2010 [13]. Women were eligible if they were of reproductive age, not pregnant and could provide informed consent. Vaginal fluid samples were collected for Gram stain, microscopy with saline and potassium hydroxide preparations, pH, and testing for STDs and other vaginal infections. Vaginal samples for molecular studies were collected using polyurethane foam swabs (Epicentre Biotechnologies, Madison,WI) brushed against the lateral vaginal wall, and stored at -80°C. BV was diagnosed for immediate management using Amsel’s criteria [6], and confirmed by Gram stain using the Nugent method [8]. Ninety-eight women (43%) had BV by Amsel’s criteria and 117 by Gram stain (53%). 

### DNA extraction, quantification and qPCR

DNA from vaginal swabs was extracted using the Ultra-Clean Soil DNA Kit or the Bacteremia Kit (Mobio, Carlsbad,CA) which gave similar results. Sham swabs without human contact were included to assess contamination from extraction reagents or collection swabs. Total bacterial load (16S-rRNA gene copies/sample) was evaluated by broad-range quantitative PCR (qPCR) with *Escherichia coli* plasmid standards ranging from 10^7^ to 10 gene copies for each reaction [13]. Concentrations of BVAB1 and *Mobiluncus* species (*Mobiluncus curtisii* and *Mobiluncus mulieris*) were assessed by bacterium-specific qPCR assays [20,21]

### Broad-range PCR and pyrosequencing of 16S rRNA gene amplicons

We performed broad-range 16S-rRNA gene PCR with pyrosequencing using 454 Life Sciences FLX technology (Roche, Branford,CT) targeting the V3-V4 region of the 16S-rRNA gene [13]. Sequence reads (426,602 reads) were classified using a phylogenetic placement tool *pplacer* [22] and a curated reference set of key vaginal bacteria [13]. Species level classification was achieved for 98·5% of reads. All sequence reads were deposited in the NCBI Short Read Archive (SRA051298) [13].

### Fluorescence in situ hybridization

Vaginal fluid smears on glass slides were fixed in 95% ethanol. Nonspecific binding was blocked using 2% sheared salmon sperm DNA in hybridization buffer without probe for 2h at 45°C. Hybridization buffer contained 5XSET (0·75M NaCl, 5mM EDTA, 0·1M Tris, pH 7·8), 10% dextran, 0·2% BSA, 0·1mg/mL polyadenosine, and 0·02% SDS. After blocking, slides were immersed in hybridization buffer with 1% sheared salmon sperm DNA and bacterium-specific probes (200ng/100µL hybridization buffer) and incubated overnight at 45°C. Probes included: broad-range-Eub-338-Cy5 (5'-GCTGCCTCCCGTAGGAGT-Cy5-3'), BVAB1-132-Fl (5'-CTGCTATCCCCCCGGTACAGG-Fl-3'), and Mobil-126-Cy3 (5'-TCCCAAAGAAAAGGACAGGTTACTC-Cy3-3'). Cells were also stained with 4',6-diamidino-2-phenylindole (DAPI) which binds to DNA. Post-hybridization, slides were washed, air dried in the dark. Bacteria were visualized using epifluorescence microscopy with a 100X oil immersion objective. Cells from 3-6 representative high-powered fields per smear were enumerated (ImageJ software) [23]. *M. curtisii* and *M. mulieris* cultures were used as control bacteria.

### Gram stains

Heat-fixed smears on slides were flooded with crystal violet for 60s, washed with tap water; flooded with iodine mordant for 60s, washed with tap water. Decolorizer comprised equal volumes of 100% ethanol and acetone. Cells were counterstained with safranin for 60s, washed with tap water. Bacteria were visualized under 100X oil immersion using bright field microscopy and enumerated using the Nugent method for BV diagnosis [8].

### Statistical analysis

The frequency matrix of sequence reads in each sample were clustered using the Dirichlet Multinomial Mixture (DMM) model [24] to evaluate overall associations between bacterial morphotypes and sequence reads. The model accommodates differences in the total number of reads per sample and the sparse taxonomic distribution, where few taxa are very abundant and many taxa are rare. DMM models were fit to the frequency matrix, with the number of distinct mixture components chosen to minimize the Laplace approximation of the model evidence. Results are displayed as a heat map, showing samples assigned to their maximum mixture component. Taxa are ordered by contribution to variation between assigned components, then grouped by genus [25].

Relationship between bacterial taxa and morphotypes was modeled using a zero-inflated generalized linear model [26]. This approach models samples with zero counts separately from samples with non-zero counts, and is appropriate for our data because number of samples with zero counts is inflated relative to the expectation obtained from samples with non-zero counts.

## Results

### Association of Gram stain morphotypes with bacterial taxa

**Figure 1:**
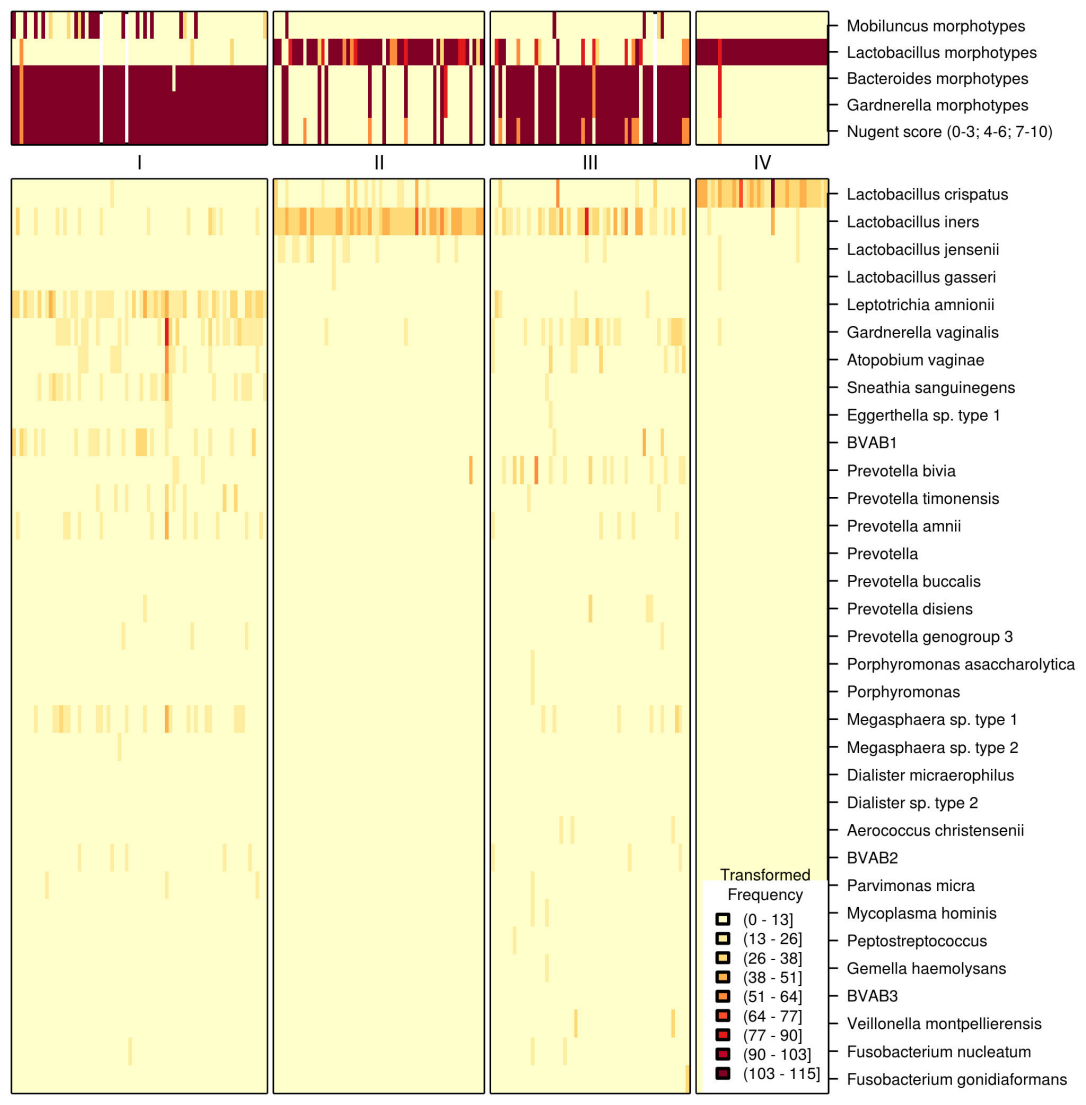
Heat map of bacterial sequence reads clustered using Dirichlet Multinomial Mixtures model. Classification and clustering of bacterial sequence reads from 220 women resulted in four clusters. Transformed frequencies of sequence reads are shown in the legend with darker colors representing more abundant sequence reads. The abundance of bacterial morphotypes, assessed by Gram stain, are reflected as numbers ranging from 0 to 4 with higher numbers indicating increased abundance. Nugent scores used for diagnosis of BV are presented as follows: BV negative (0-3), Intermediate score (4-6) and BV positive (7-10). White bars denote that data are not available. The model was not informed of bacterial morphotype data. Taxa are ordered based on contribution to variance between clusters, and then are grouped by genus-level classifications. The heat map includes taxa whose total abundance across samples exceeds the 80^th^ quantile. Note absence of *Mobiluncus* species in the list of taxa presented due to low abundance of sequence reads.

Classified 16S-rRNA gene sequence reads from vaginal samples clustered into four groups using the Dirichlet Multinomial Mixture model (DMM) (Figure 1). Cluster-I included vaginal samples from women whose bacterial communities were similar to those observed in BV. Morphotypes by Gram stain in Cluster-I included *Gardnerella* and *Bacteroides*, with Lactobacillus morphotypes absent. Cluster-II was dominated by *L. iners* reads and a high abundance of Lactobacillus morphotypes. Cluster-III contained mixed microbiota by broad-range PCR/pyrosequencing, with *L. iners* and low abundance of BV-associated bacteria reads including *G. vaginalis* and *Prevotella bivia*; most of these samples contained *Gardnerella* and *Bacteroides* morphotypes by Gram stain (49/55). *G. vaginalis* reads were present in 53/55 samples (Rank abundance median, 6·6%) and *Bacteroides* reads were either absent or rare. Although *L. iners* reads were present in 47/55 samples in Cluster-III (Rank abundance median, 17·7%), only 16/55 samples were noted to have Lactobacillus morphotypes. Cluster-IV was dominated with *L. crispatus* by broad-range PCR/pyrosequencing; all samples had Lactobacillus morphotypes.

**Figure 2:**
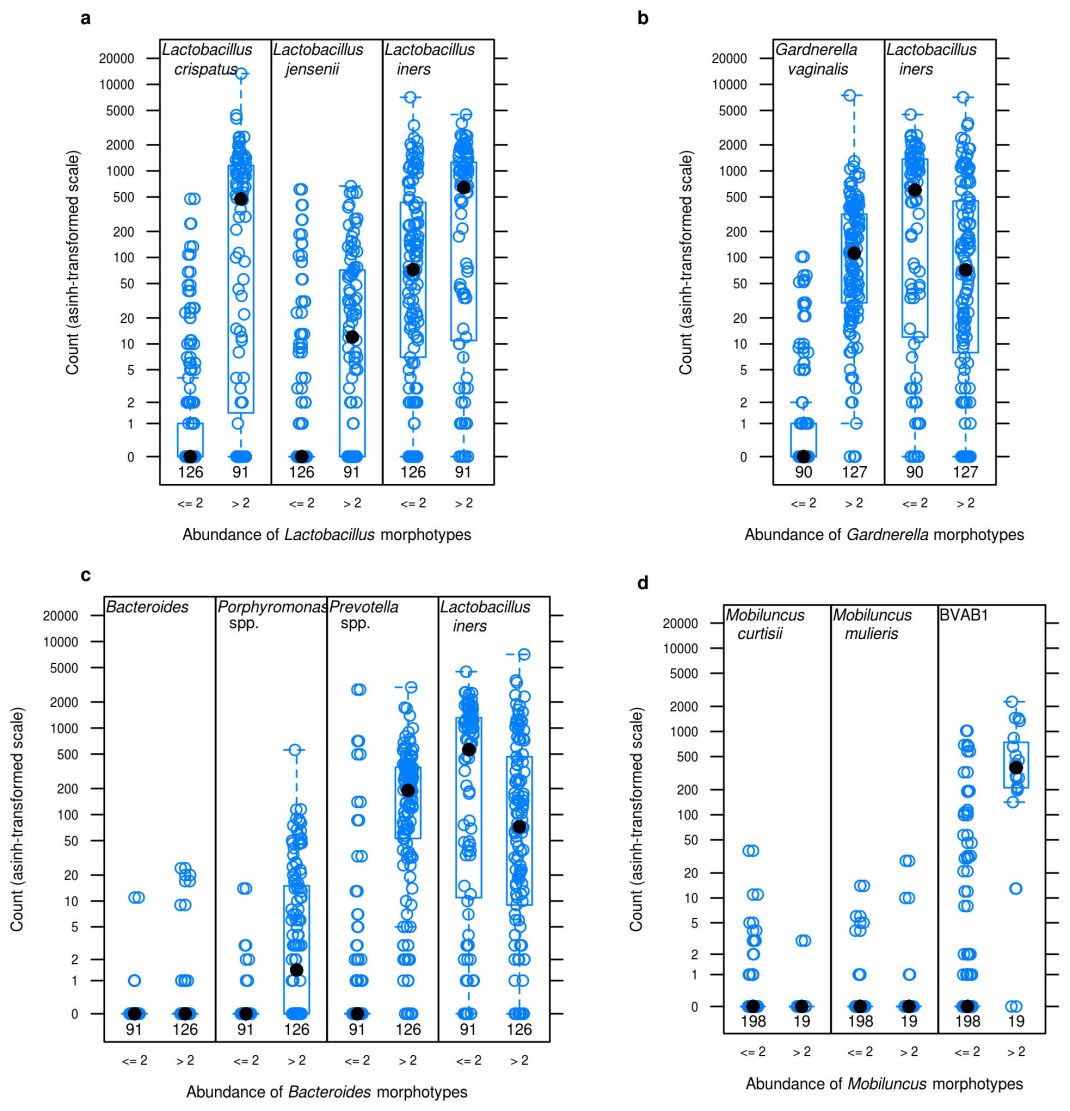
Association of bacterial sequence reads with Gram stain bacterial morphotypes. Based on *a* *priori* hypothesis and abundance of taxa represented in DMM clustering, bacterial sequence reads (asinh-transformed) obtained using broad-range PCR and pyrosequencing (Y-axis) were correlated with abundance of Gram stain morphotypes (X-axis). The inverse hyperbolic sin (asinh) is a log-like transformation with better (linear) behavior near zero. This makes it applicable to count data with many zeros, as is found in our data. Data are grouped as <=2 (0, 1+, 2+) and >2 (3+, 4+) (X-axis) which indicate average number of bacterial morphotypes observed under oil-immersion per high-powered field. 0, no morphotypes present; 1+, <1 morphotype present; 2+, 1 to 4 morphotypes present; 3+, 5 to 30 morphotypes present; 4+, 30 or more morphotypes present. Open blue circles denote abundance of sequence reads from a single woman; closed black circles indicate median values. Numbers below box plots denote numbers of samples in each group.

**Figure 3:**
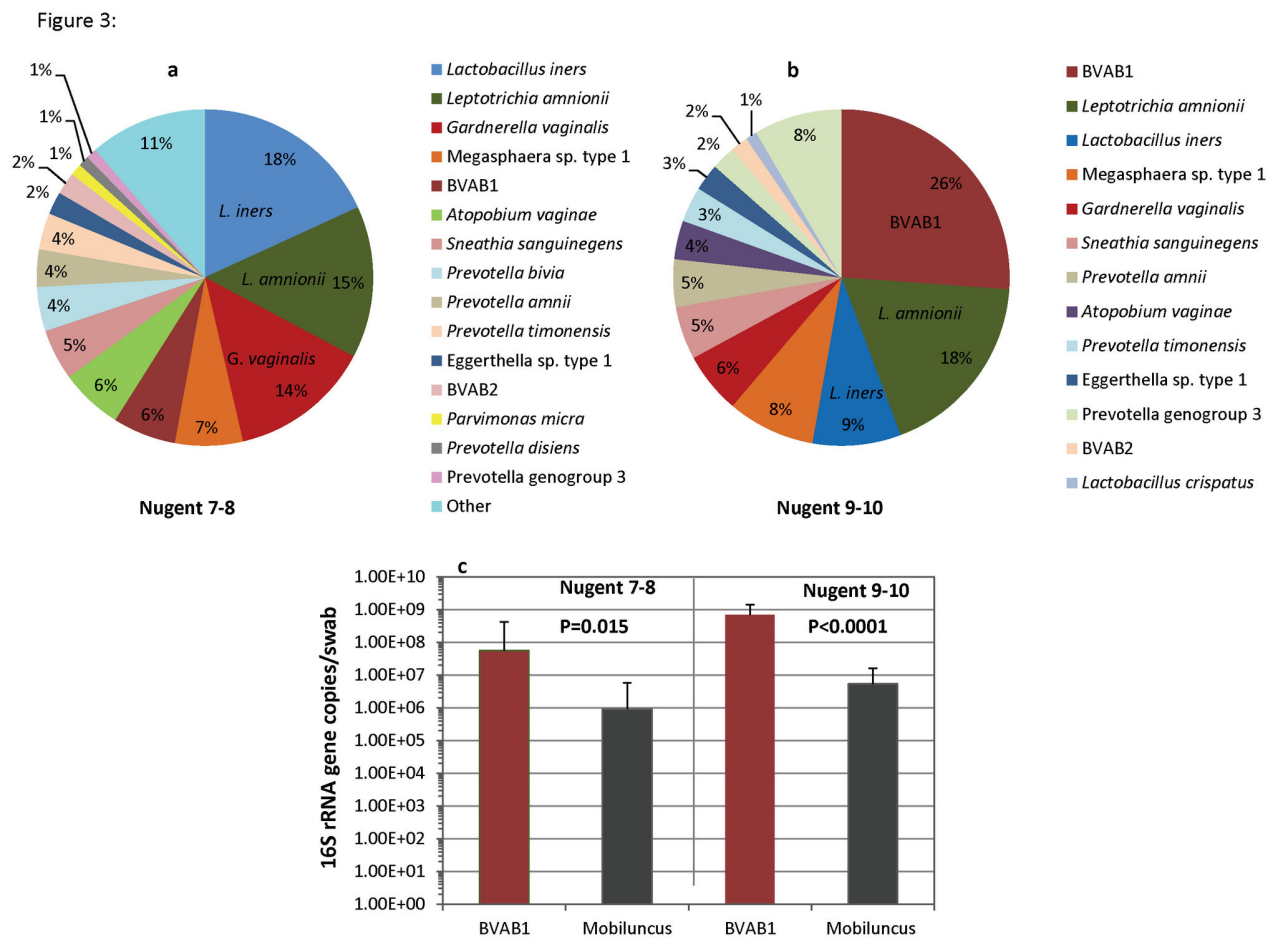
Curved Gram negative rods are more likely to be BVAB1 rather than *Mobiluncus* species. Comparison of rank abundance plots in women with Nugent scores of 7-8 (A) and 9-10 (B) show percentage of sequence reads in each group. Women with Nugent scores 9-10 are dominated with BVAB1. Quantitative PCR targeting BVAB1 and *Mobiluncus* (C) was performed for vaginal samples obtained from women with BV demonstrating higher concentrations of BVAB1 compared to *Mobiluncus* in each group.

Sequence reads classified as BVAB1 were associated with *Mobiluncus* morphotypes on Gram stain (Figure 2, Table 1). Most strikingly, *Mobiluncus curtisii* sequence reads were not associated with presence of *Mobiluncus* morphotypes. Although there was a significant association between presence of *Mobiluncus mulieris* sequences and *Mobiluncus* morphotypes, this species was sparsely represented; only 9/220 samples contained *M. mulieris* reads at 0·18% median relative abundance, and median values were not different between high morphotype abundance and low abundance groups (Figure 2). In contrast, BVAB1 reads were present in 47 samples (21·4%) with a relative abundance of 0·02%-94% (Median, 8·8%). Rank abundance plots of vaginal bacteria in women with curved rods by Gram stain (Nugent scores 9-10) showed that BVAB1 was the dominant bacterium (26·1%) while relative abundance of *M. curtisii* and *M. mulieris* was 0·12% and 0·09% respectively (Figure 3). Presence of *Lactobacillus* reads was significantly associated with Lactobacillus morphotypes, as expected (Figure 2, Table 1). *L. crispatus* and *L. jensenii* reads were not detected in 70% and 82% of women whose samples had low abundance of Lactobacillus morphotypes and this was statistically significant (Table 1). In contrast, *L. iners* reads were absent in only 13% of women whose samples had low abundance of Lactobacillus morphotypes, suggesting that while *L. iners* can contribute to *Lactobacillus* morphotypes, it may be associated with other Gram stain morphotypes or present at lower concentrations. Samples with *Gardnerella* and *Bacteroides* morphotypes (scored separately in our analysis) were highly concordant (99·5%) (Figure 1). Although presence of *Bacteroides* reads was significantly associated with presence of *Bacteroides* morphotypes, *Bacteroides* reads were infrequent with a relative abundance of 0·009% in women with BV, hence unlikely to contribute to *Bacteroides* morphotypes seen in Gram stains. In contrast, *Prevotella* and *Porphyromonas* sequence reads were highly associated with *Bacteroides* morphotypes, and were more abundant (Figure 2, Table 1).

**Table 1:** Statistical association of bacterial sequence reads with Gram stain morphotypes.

**Lactobacillus morphotypes**			
**Proportion of zero counts^1^**	**<=2^2^**	**>2^2^**	**P-value^3^**
*Lactobacillus crispatus*	0·7	0·242	**p<0·0001**
*Lactobacillus jensenii*	0·82	0·308	**p<0·0001**
*Lactobacillus iners*	0·13	0·077	2·4E-01
**Average of non-zero counts**	**<=2**	**>2**	**P-value**
*Lactobacillus crispatus*	34	1108	**p<0·0001**
*Lactobacillus jensenii*	88	106	**p<0·0001**
*Lactobacillus iners*	486	887	**p<0·0001**
***Gardnerella* morphotypes**			
**Proportion of zero counts**	**<=2**	**>2**	**P-value**
*Gardnerella vaginalis*	0·7	0·024	**p<0·0001**
*Lactobacillus iners*	0·078	0·126	2·6E-01
**Average of non-zero counts**	**<=2**	**>2**	**P-value**
*Gardnerella vaginalis*	16	270	**p<0·0001**
*Lactobacillus iners*	870	502	**p<0·0001**
***Bacteroides* morphotypes**			
**Proportion of zero counts**	**<=2**	**>2**	**P-value**
*Bacteroides*	0·96	0·94	5·8E-01
*Porphyromonas*	0·96	0·47	**p<0·0001**
*Prevotella*	0·76	0·04	**p<0·0001**
*Lactobacillus iners*	0·088	0·12	4·6E-01
**Average of non-zero counts**	**<=2**	**>2**	**P-value**
*Bacteroides*	3·5	9·2	**1·1E-03**
*Porphyromonas*	5	34·2	**p<0·0001**
*Prevotella*	195	292·1	**p<0·0001**
*Lactobacillus iners*	870·2	502·1	**p<0·0001**
***Mobiluncus* morphotypes**			
**Proportion of zero counts**	**<=2**	**>2**	**P-value**
*Mobiluncus curtisii*	0·95	0·947	9·20E-01
*Mobiluncus mulieris*	0·97	0·842	**1·80E-02**
BVAB1	0·86	0·053	**p<0·0001**
**Average of non-zero counts**	**<=2**	**>2**	**P-value**
*Mobiluncus curtisii*	6·8	3	1·7E-01
*Mobiluncus mulieris*	5·2	13	**p<0·0001**
BVAB1	150·9	631	**p<0·0001**

1 Counts refer to bacterial sequence reads that were asinh-transformed.

2 <=2 (0, 1+, 2+) and >2 (3+, 4+) refer to abundance of bacterial morphotypes described by Nugent et al. and used in the weighted standardized scoring system to generate Nugent scores for Gram stains (8).

3 Significant P-values are shown in boldface type.

### Quantitative PCR targeting BVAB1 and *Mobiluncus*

To ensure that we could reliably detect and measure concentrations of BVAB1 and *Mobiluncus*, we performed taxon-directed qPCR in all women with BV. Among women with Nugent scores 9-10, mean concentration of BVAB1 DNA was 2-log units greater than *Mobiluncus* (Figure 3C) (p<0·0001). Among women with Nugent scores 7-8, *Mobiluncus* and BVAB1 were detected by qPCR in 41% and 19%, with mean concentrations of 9·2×10^5^ and 5·6×10^7^ copies respectively, although no curved rods were detected by Gram staining. Comparison of results from broad-range PCR/pyrosequencing and qPCR showed that there was 94% concordance for BVAB1, but only 52% for *Mobiluncus*. Quantitative PCR can detect low levels of *Mobiluncus* DNA (1·9×10^3^ copies/swab, lowest level detected), which falls below the detection limit of the broad-range PCR/pyrosequencing approach.

### FISH targeting BVAB1 and *Mobiluncus*

**Figure 4:**
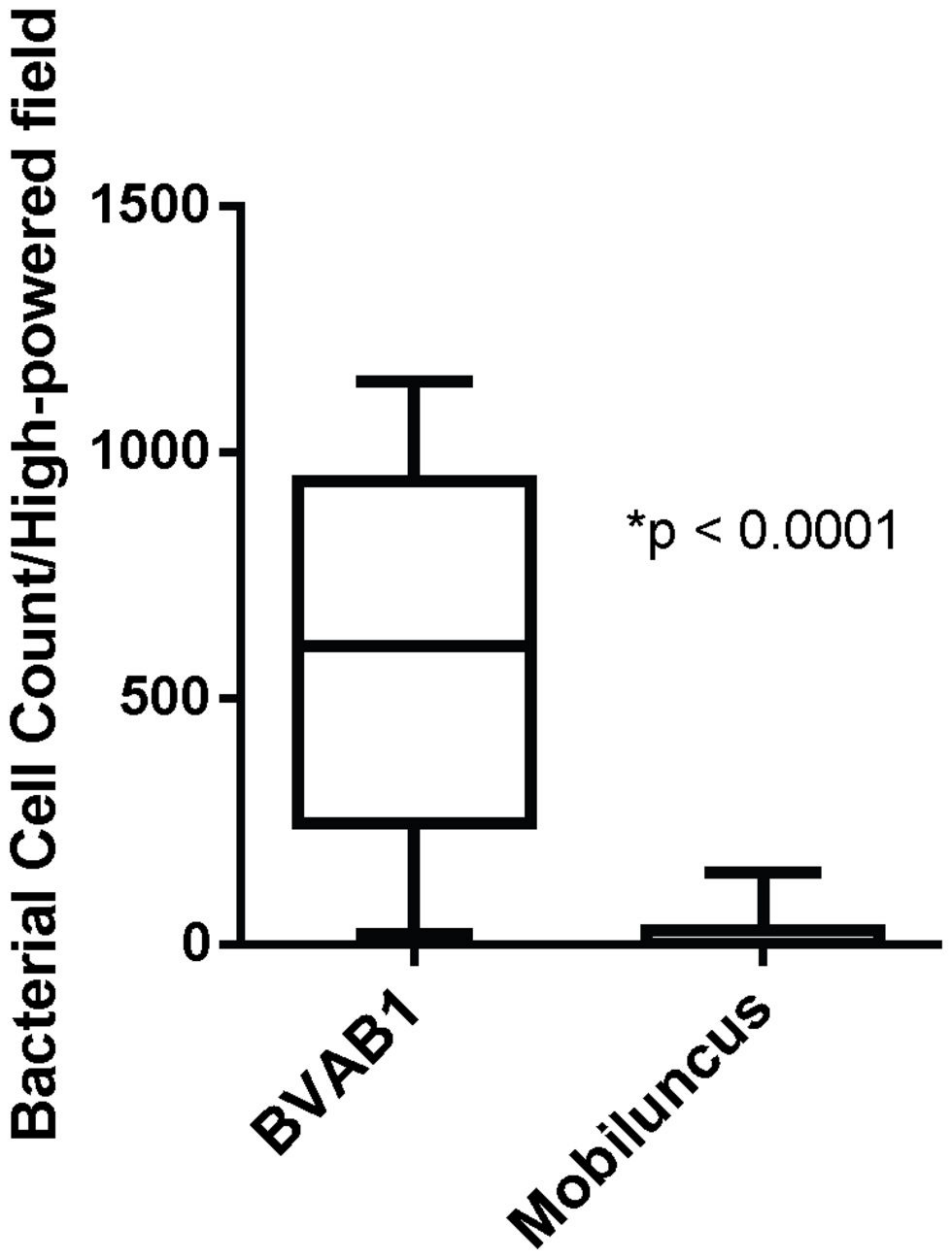
FISH reveals higher quantities of BVAB1 cells than *Mobiluncus* cells in vaginal fluid. Vaginal fluid smears from all women with Nugent 10 were assessed for relative quantities of BVAB1 and *Mobiluncus* cells. The lines in the box plot represent the mean and whiskers denote 95% confidence intervals.

**Figure 5:**
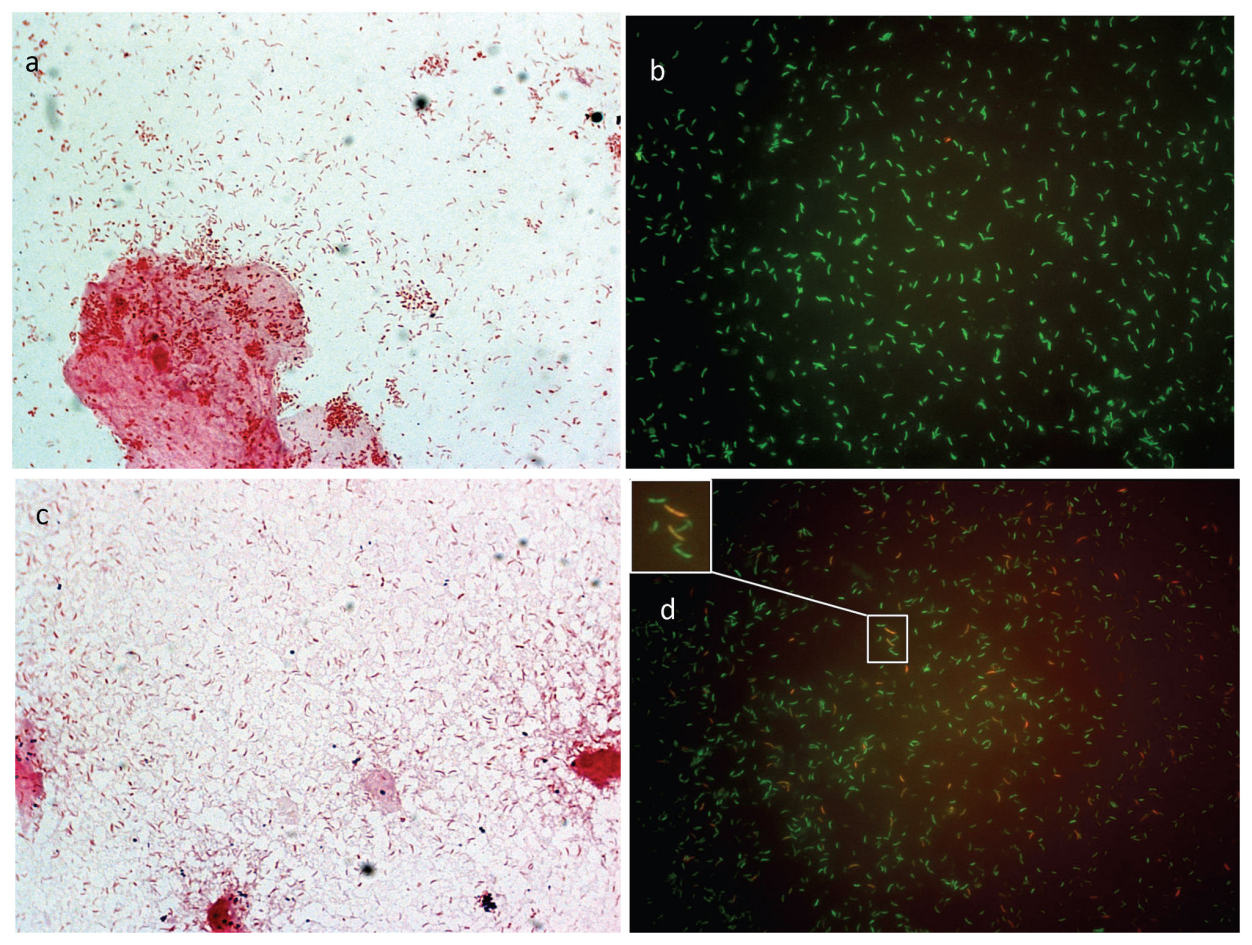
Fluorescence micrographs and Gram stain images of vaginal fluid smears. Vaginal fluid smears from two representative study participants are shown. 4+ curved rods were documented by Gram stain (A & C) for both participants who had BV (Nugent score 10). Panels A (Gram stain) and B (FISH) are vaginal fluid smears from a representative participant with low concentrations of *Mobiluncus* DNA (2·5×10^5^ copies 16S-rRNA gene/swab) and high concentrations of BVAB1 DNA (2·4×10^9^ 16S rRNA gene copies/swab). Panel B shows a field of bacteria hybridizing with probes for BVAB1 (green) while no hybridization was observed with *Mobiluncus* probe (red). Mean quantity of BVAB1 cells was 661 versus <1 *Mobiluncus* cell. Panels C (Gram stain) and D (FISH) are vaginal fluid smears from a representative participant with high concentrations of *Mobiluncus* DNA (1·3×10^7^ 16S-rRNA gene copies/swab) and BVAB1 DNA (5·1×10^8^ 16S-rRNA gene copies/swab). Panel D shows a field of bacteria hybridizing with both *Mobiluncus* (red) and BVAB1 (green) DNA. Mean quantity of BVAB1 cells was 908 versus 145 *Mobiluncus* cells.

We visualized BVAB1 and *Mobiluncus* by FISH in all samples with Nugent scores of 10 to assess relative quantities using a third approach. In 16/17 samples with swabs available, mean numbers of BVAB1 cells from representative images from each sample were significantly higher than *Mobiluncus* cells (p<0·0001, Figures 4, 5). In 1/17 samples, bacterial cells were visible using DAPI staining, but no fluorescence was obtained with BVAB1 or *Mobiluncus* probes. However, there was also a lack of fluorescence with Eub338 (broad-range probe), suggesting there was degradation of RNA and resulting loss of fluorescence in this sample. 

## Discussion

The gold standard for BV diagnosis or confirmation is Gram staining of vaginal fluid and interpretation with a weighted scoring system based on abundance of bacterial morphotypes [7,8]. Identity of morphotypes has largely been inferred from cultivation-based studies [27,28]. Since the publication of the Nugent scheme in 1991 [8], several uncultivated bacteria have been shown to be highly specific for BV such as BVAB1 [10]. Here, we systematically examined associations of bacteria detected using high-throughput sequencing methods with individual components of the Nugent score including Lactobacillus, *Gardnerella*, *Bacteroides*, and *Mobiluncus* morphotypes. Our findings suggest that *Bacteroides* and *Mobiluncus* morphotypes do not accurately reflect the presence of these bacteria in the human vagina.

DMM clustering resulted in identification of four community clusters based on distribution of 16S rRNA gene sequence reads (Figure 1). High abundance of reads classified as *L. crispatus* and *L. iners* were concordant with Lactobacillus morphotypes. The correlation between Lactobacillus morphotypes and lower abundance of *L. iners* was not apparent, and it may be that cell numbers of *L. iners* are sufficiently infrequent such that Lactobacillus morphotypes are not reported in the Nugent score. Many women in Cluster-III are diagnosed as having BV by Gram stain despite presence of *L. iners*, highlighting the fact that BV is not synonymous with absence of all lactobacilli. To address differences in Lactobacillus morphotypes, Verhelst et al. presented a refined Ison and Hay scoring method for Gram stain to distinguish additional grades within the *Lactobacillus*-dominated microbiota [29]. 

A critical distinction between women with Nugent scores of 7-8 and those with scores of 9-10 is presence of curved Gram-negative rods in the latter group. *Mobiluncus* has been cultivated from vaginal samples with Nugent scores 9-10, leading to the association between *Mobiluncus* and curved Gram-negative rod morphotypes [27,30]. A striking observation from our broad-range PCR and pyrosequencing data was the high relative abundance of BVAB1 reads in women with Nugent scores 9-10 (26%) compared to 100-fold lower abundance of *Mobiluncus* reads (0·2%) (Figure 3B). Statistical analysis revealed that curved rod morphotypes were associated with BVAB1 (p<0.001), but not with *Mobiluncus*. These data suggested the hypothesis that curved Gram-negative rods observed on Gram stains of vaginal fluid and designated “Mobiluncus morphotypes” are likely to be BVAB1. 

We tested this hypothesis using independent methods. It is possible that the V3-V4 region of the 16S rRNA gene selected in our study is not optimal for the amplification of *Mobiluncus* despite complete homology in our primer target sequence. Hummelen et al. characterized vaginal bacterial communities by targeting the V6 region of the 16S rRNA gene [11]. They showed that an uncultured Lachnospiraceae bacterium (100% identical to BVAB1) was present at >1% relative abundance in 73% of women with BV, and *Mobiluncus* was below the 1% relative abundance threshold. Given that the Hummelen study targeted a different region of the 16S rRNA gene, it is unlikely that amplification bias accounts for low numbers of *Mobiluncus* sequences in these studies. 

We determined if BVAB1 DNA concentrations were higher than *Mobiluncus* DNA by using species-specific qPCR in all women with BV (Figure 3C). BVAB1 was detected in all women with Nugent scores 9-10, and median concentration of BVAB1 DNA was significantly greater than concentration of *Mobiluncus*. In contrast, *Mobiluncus* DNA was detected in 76% of women with Nugent scores of 9-10, and median concentration was 3-log units less compared to BVAB1 DNA. The qPCR data are concordant with the broad-range PCR/pyrosequencing data showing that BVAB1 is more prevalent and found in higher abundance than *Mobiluncus* in women with Nugent scores 9-10. Our results are substantiated by observations made by Zozaya-Hinchliffe et al. using qPCR, wherein the investigators showed elevated quantities of BVAB1 (>10^7^ copies) in 89% of women with Nugent scores 9-10, while concentrations of *Mobiluncus* were much lower (10^1^-10^2^ copies); this observation was not emphasized in that study [31].

Bacteria can have different numbers of rRNA gene operons/genome, and the exact number is unknown for many uncultivated organisms [32]. *Mobiluncus curtisii* ATCC43063 has two 16S rRNA gene copies per genome [33]. The exact number of 16S rRNA gene copies per genome for BVAB1 is currently not available. One challenge for correlating 16S rRNA gene copies to absolute quantities of bacteria is that bacteria with high copy numbers/genome may be over-represented using PCR-based approaches. To overcome copy number bias, we performed FISH to quantify BVAB1 and *Mobiluncus* cells in vaginal fluid from women with Nugent scores 10 (Figures 4, 5). Greater numbers of BVAB1 were detected by FISH (p<0·0001) which confirmed our observations from qPCR and broad-range PCR with pyrosequencing. FISH provided direct microscopic evidence that BVAB1 is more abundant than *Mobiluncus*, and therefore is more likely contributing to curved Gram-negative rods. Furthermore, the abundance of BVAB1 cells on FISH closely matches the abundance of curved Gram-negative rods seen with Gram stain itself (Figure 5). Together, these separate lines of investigation suggest that curved Gram-negative rods seen on Gram stain of vaginal fluid in women with BV are more likely to be BVAB1 than *Mobiluncus*.

*Bacteroides* morphotypes were previously linked to *Bacteroides* species based on cultivation studies [34]. Since then, changes in taxonomy of the *Bacteroides fragilis* group based on phylogeny and biochemical properties have led to re-classification of many of these bacteria as *Prevotella* and *Porphyromonas*. The genus *Bacteroides* now contains only bile-resistant Gram-negative rods, while the saccharolytic, bile-sensitive pigmented and non-pigmented species were re-classified to the genus *Prevotella* and the pigmented asaccharolytic species to the genus *Porphyromonas* [35-38]. Although current evidence suggests that there is no association of the *Bacteroides fragilis* group with BV [39], there continue to be studies investigating this link [18]. In our analysis as well, *Bacteroides* reads were uncommon in women with high abundance of *Bacteroides* morphotypes (Figure 2). Instead, we found that *Prevotella* and *Porphyromonas* were significantly associated with the *Bacteroides* morphotype (Table 1). Hummelen et al. also did not report high abundance of *Bacteroides*, but noted high abundance of *Prevotella* and *Porphyromonas* in women with BV [11]. Cultivation studies may detect *Bacteroides* despite being present at low abundance because these bacteria are readily cultivable. Although we scored for *Gardnerella* and *Bacteroides* morphotypes separately in our study, it can be difficult to consistently differentiate between them by Gram stain [8]. A limitation in our analysis of associations between bacteria detected by PCR and *Gardnerella* or *Bacteroides* morphotypes is this high level of correlation for these two bacterial types, restricting our ability to link sequence reads to one of these morphotypes. 

In conclusion, we systematically examined correlations between vaginal bacterial species described by broad-range PCR and high-throughput sequencing with individual components of the Gram stain Nugent score. Curved Gram-negative rods observed by Gram stain are more likely to be the uncultivated bacterium, BVAB1, rather than the widely accepted *Mobiluncus* species. *Prevotella* and *Porphyromonas* are likely major contributors to *Bacteroides* morphotypes, whereas *Bacteroides* species are infrequent. Different bacterial communities with similar Gram stain characteristics may impart different risks for complications associated with BV, highlighting the importance of distinguishing between these bacterial communities using modern molecular approaches. In the future, it would be more accurate to list the bacterial morphotypes in the Nugent score as Lactobacillus, Gardnerella/Prevotella/Porphyromonas, and BVAB1/Mobiluncus morphotypes. 
